# Stability of Pharmaceutical Co-Crystals at Humid Conditions Can Be Predicted

**DOI:** 10.3390/pharmaceutics13030433

**Published:** 2021-03-23

**Authors:** Heiner Veith, Maximilian Zaeh, Christian Luebbert, Naír Rodríguez-Hornedo, Gabriele Sadowski

**Affiliations:** 1 Laboratory of Thermodynamics, Department of Chemical and Biochemical Engineering, TU Dortmund University, Emil-Figge-Str. 70, D-44227 Dortmund, Germany; heiner.veith@tu-dortmund.de (H.V.); maximilian.zaeh@tu-dortmund.de (M.Z.); christian.luebbert@tu-dortmund.de (C.L.); 2 Department of Pharmaceutical Sciences, University of Michigan, 428 Church St, Ann Arbor, MI 48109-1065, USA; nrh@med.umich.edu

**Keywords:** cocrystal, disproportionation, stability, deliquescence, moisture sorption, PC-SAFT, phase diagrams, relative humidity

## Abstract

Knowledge of the stability of pharmaceutical formulations against relative humidity (RH) is essential if they are to become pharmaceutical products. The increasing interest in formulating active pharmaceutical ingredients as stable co-crystals (CCs) triggers the need for fast and reliable in-silico predictions of CC stability as a function of RH. CC storage at elevated RH can lead to deliquescence, which leads to CC dissolution and possible transformation to less soluble solid-state forms. In this work, the deliquescence RHs of the CCs succinic acid/nicotinamide, carbamazepine/nicotinamide, theophylline/citric acid, and urea/glutaric acid were predicted using the Perturbed-Chain Statistical Associating Fluid Theory (PC-SAFT). These deliquescence RH values together with predicted phase diagrams of CCs in water were used to determine critical storage conditions, that could lead to CC instability, that is, CC dissolution and precipitation of its components. The importance of CC phase purity on RH conditions for CC stability is demonstrated, where trace levels of a separate phase of active pharmaceutical ingredient or of coformer can significantly decrease the deliquescence RH. The use of additional excipients such as fructose or xylitol was predicted to decrease the deliquescence RH even further. All predictions were successfully validated by stability measurements at 58%, 76%, 86%, 93%, and 98% RH and 25 °C.

## 1. Introduction

In a pharmaceutical co-crystal (CC), the active pharmaceutical ingredient (API) and the coformer (CF) are arranged in a common crystal lattice [1]. The CC might have significantly improved physical properties compared to the pure API, such as increased aqueous solubility or stability [2,3]. Due to the change in the regulatory framework of authorities such as the Food and Drug Administration (FDA) [4] regarding the intellectual property and impact on the product-lifecycle-management, CCs have gained increasing interest in recent years [5]. An important factor for the approval of CCs is their stability against environmental changes or stress conditions [6,7,8].

CCs have been reported to transform to API or CF solid phases, hydrates, and polymorphs upon exposure to different levels of relative humidity (RH) [8,9,10]. The acetylsalicylic acid/acetamide CC was shown to undergo conversions at RH conditions as low as 75% RH already after four days [11]. Nevertheless, other CCs, e.g., the theophylline/oxalic acid CC are stable even at 98% RH for 49 days, although the pure theophylline would transform to its hydrate within one day at that RH [9]. Thakuria et al. [6] found that the transformation of the caffeine/glutaric acid CC to the caffeine hydrate at 98% RH is a stepwise process. First, the CC deliquesces (liquid phase evolves with the dissolved CC components) and afterwards, the caffeine hydrate crystallizes from the liquid phase. Thus, a critical quality attribute with respect to stability of the CC is the deliquescence RH (DRH).

To prevent time-consuming and expensive stability measurements, Eddleston et al. [12] developed an experimental evaluation of the CC stability at high RH conditions based on the addition of liquid water to a stoichiometric mixture of the CC components. With this method they investigated, if the phase behavior of the CC in water is congruent or incongruent [12]. The mechanism by which moisture sorption leads to CC formation was determined by Jayasankar et al. [13]. It was shown that CCs of carbamazepine/nicotinamide, carbamazepine/saccharin, and caffeine or theophylline with dicarboxilyc acid coformers were generated when solid mixtures of CC components deliquesce. Dissolution of CC components in the deliquesced solution led to supersaturation with respect to CCs and precipitation, even when the phase behavior of these CCs in water was incongruent. CC formation was enhanced by deliquescent formulation additives, such as sugars (fructose and sucrose), and by moisture sorption by polymers (PVP), Good et al. [14]. The role of critical water activity (equivalent to the critical RH) and CF activity on CC stability has been reported for the case of CFs and additives that modulate water activity, Jayasankar et al. [15]. CC phase purity was found to be important not only due to its influence on supersaturation, but trace levels of water-soluble CFs can significantly change the critical water activity of the sample and therewith the CC stability [15].

The prediction of critical water activities such as DRH was already investigated for single crystals and crystal mixtures in a previous work [16]. This work proposes a thermodynamic in-silico approach to predict the stability of CCs against conversions as a function of moisture uptake. This allows determining the critical RH up to which the CC is stable and predicting the influence of trace level impurities of API, CF, or additional excipients on this critical RH.

## 2. Theory

The thermodynamic equilibria between the solid phase (CC), the vapor phase (RH), and the liquid phase (present upon deliquescence and CC instability and transformation) must be considered to understand the RH influence on CCs. The solid–liquid equilibrium must be calculated for each present crystalline component (e.g., CC and CF) simultaneously. The vapor–liquid equilibrium must be solved for water being the only component present in the vapor phase.

### 2.1. Solid–Liquid Equilibrium

The equilibrium between a solid crystal (API or CF) and a liquid is fulfilled when the chemical potential of the components is equal in the solid and in the liquid phase. This is expressed by the following equation:

(1) $$x_{i}\cdot \gamma_{i}=a_{i}=\exp\left[-\frac{\Delta h_{i}^{SL}}{R\cdot T}\cdot \left(1-\frac{T}{T_{i}^{SL}}\right)-\frac{\Delta c_{p,i}^{SL}}{R}\cdot \left(\ln\left(\frac{T_{i}^{SL}}{T}\right)-\frac{T_{i}^{SL}}{T}+1\right)\right]$$

The resulting mole-fraction solubility of the component *i* ($x_{i}$) is calculated from the melting properties (melting temperature $T_{i}^{SL}$, melting enthalpy $\Delta h_{i}^{SL}$, and heat capacity difference between solid and liquid component *i* $\Delta c_{p,i}^{SL}$) and from the activity coefficient $\gamma_{i}$. R is the ideal gas constant and T is the system temperature. $\gamma_{i}$ explicitly considers interactions such as van der Waals forces and hydrogen-bond formation between all components present in the liquid phase. At solid–liquid equilibrium conditions, the thermodynamic activity $a_{i}$ of a given component *i* only depends on T.

The CC formation is considered similarly to a reaction and the solid–liquid equilibrium is described by the solubility product, which is derived considering the dissolution process of the CC in a solvent [17,18]:

(2) $$K_{s,CC}=\underset{i}{\prod }{\left(a_{i}\right)}^{\nu_{i}}={\left(x_{API}\cdot \gamma_{API}\right)}^{\nu_{API}}\cdot {\left(x_{CF}\cdot \gamma_{CF}\right)}^{\nu_{CF}}$$

The mole fractions of the API $x_{API}$ and the CF $x_{CF}$ indicating the CC solubility are calculated using the activity coefficients of each component and the stoichiometry of the CC ($\nu_{API}$, $\nu_{CF}$). The CC-specific and solvent-independent CC solubility product ($K_{s,CC}$) was determined in this work via fitting to the solubility of the CC in one solvent. Afterwards, it was used to predict the solubility in water.

The solid–liquid equilibrium for a hydrate (of the API or CF) is again calculated using the solubility product, which is derived from the dissolution process in water [16,19]:

(3) $$K_{s,hydrate}=\underset{k}{\prod }{\left(a_{k}\right)}^{\nu_{k}}={\left(x_{i}\cdot \gamma_{i}\right)}^{\nu_{i}}\cdot {\left(x_{water}\cdot \gamma_{water}\right)}^{\nu_{water}}$$

The resulting solubility of the hydrate $x_{i}$ depends on the water mole fraction $x_{water}$, the activity coefficients of water and of the hydrate-forming component i and the stoichiometry of the hydrate ($\nu_{i}$, $\nu_{water}$). The solubility product of the hydrate $K_{s,hydrate}$ is determined by the following equation:

(4) $$K_{s,hydrate}={\left(exp\left[-\frac{\Delta h_{i}^{SL}}{R\cdot T}\left(1-\frac{T}{T_{i}^{SL}}\right)-\frac{\Delta c_{P,i}^{SL}}{R}\left(\ln\left(\frac{T_{i}^{SL}}{T}\right)-\frac{T_{i}^{SL}}{T}+1\right)\right]\right)}^{{}_{i}}\;\cdot \;{\left(a^{trans}\cdot exp\left[\frac{-\Delta h^{trans}}{\nu_{water}\cdot R\cdot T}\left(1-\frac{T}{T^{trans}}\right)\right]\right)}^{\nu_{water}}$$

The solubility product of the hydrate depends on the melting properties of the component *i* (API or CF) and the hydrate/anhydrate transition properties ($T^{trans}$ is the temperature, above which the hydrate is not thermodynamically stable anymore, $\Delta h^{trans}$ is the enthalpy upon dehydration, and $a^{trans}$ is the transition water activity at which hydrate and anhydrate are in equilibrium).

The solid–liquid equilibrium of a CC hydrate is calculated using a solubility product representing the dissolution process in water:

(5) $$K_{s,CC\;hydrate}=\underset{k}{\prod }{\left(a_{k}\right)}^{\nu_{k}}={\left(x_{API}\cdot \gamma_{API}\right)}^{\nu_{API}}\cdot {\left(x_{CF}\cdot \gamma_{CF}\right)}^{\nu_{CF}}\cdot {\left(x_{water}\cdot \gamma_{water}\right)}^{\nu_{water}}$$

The solubility product was determined in this work from the solubility of the CC hydrate in water.

### 2.2. Vapor-Liquid Equilibrium

For the calculation of the vapor–liquid equilibrium it is assumed that the vapor phase behaves like an ideal gas:

(6) $$x_{water}\cdot \gamma_{water}=a_{water}=\frac{p_{water}}{p_{0,water}^{LV}}=\frac{RH}{100\%}$$

Therefore, the water activity in the liquid phase equals RH. The RH is defined as the partial pressure of water $p_{water}$ divided by the saturation vapor pressure of water $p_{0,water}^{LV}$. The equilibrium mole fraction of water in the liquid phase is determined by its activity coefficient $\gamma_{water}$ which depends on all components present in the liquid as well as on their concentrations.

### 2.3. Deliquescence

DRH is used to determine the stability of CCs and CC hydrates. It is assumed that the CC formation or transformation into its solid components occurs via a dissolution mediated process in the deliquesced liquid phase [13,14,15,20]. The DRH is calculated by solving the solid–liquid–vapor equilibrium of the present components. The solid–liquid equilibrium must be solved simultaneously for each crystalline component present (Equations (1), (2), (3), or (5)), the vapor–liquid equilibrium (Equation (6)) must be fulfilled at the composition of the saturated liquid phase regarding all crystalline components.

### 2.4. PC-SAFT

All interactions between the components in the liquid phase are considered by the activity coefficients. The activity coefficients were calculated in this work from the residual Helmholtz energy $A^{residual}$ obtained from the Perturbed-chain statistical associating fluid theory (PC-SAFT) [21,22]. In this work, $A^{residual}$ is summed up from three contributions: $A^{hard-chain}$ considers the hard-chain repulsions, $A^{dispersion}$ considers the attractions like van der Waals forces, and $A^{association}$ considers the hydrogen bond formation:

(7) $$A^{residual}=A^{hard-chain}+A^{dispersion}+A^{association}$$

Every molecule is described as a chain of $m_{seg}$ segments with the segment diameter $\sigma_{i}$, the dispersion-energy parameter $u_{i}/k_{B}$, the association-energy parameter $\epsilon^{A_{i}B_{i}}/k_{B}$, and the association volume $\kappa^{AiBi}$. Berthelot–Lorentz [23,24] mixing rules were used to obtain the segment diameter and dispersion energy in mixtures of components *i* and *j*:

(8) $$\sigma_{ij}=\frac{1}{2}\left(\sigma_{i}+\sigma_{j}\right)$$

(9) $$u_{ij}=\sqrt{u_{i}u_{j}}\cdot \left(1-k_{ij}\right)$$

The dispersion energy may be corrected by a binary interaction parameter $k_{ij}$ to better describe experimental data of the binary mixture. The $k_{ij}$ may be temperature dependent with a slope of $k_{ij,T}$ and a reference $k_{ij,b}$ at 0 K.

(10) $$k_{ij}=k_{ij,T}\cdot T\left[K\right]+k_{ij,b}$$

The mixing rules of Wolbach and Sandler [25] were used to determine the association energy and association volume of mixtures:

(11) $$\varepsilon^{AiBj}=\frac{1}{2}\left(\varepsilon^{AiBi}+\varepsilon^{AjBj}\right)$$

(12) $$\kappa^{AiBj}=\sqrt{\kappa^{AiBi}\kappa^{AjBj}}{\left(\frac{\sqrt{\sigma_{i}\sigma_{j}}}{\left(1/2\right)\left(\sigma_{i}+\sigma_{j}\right)}\right)}^{3}$$

## 3. Phase Diagrams

**Figure 1:**
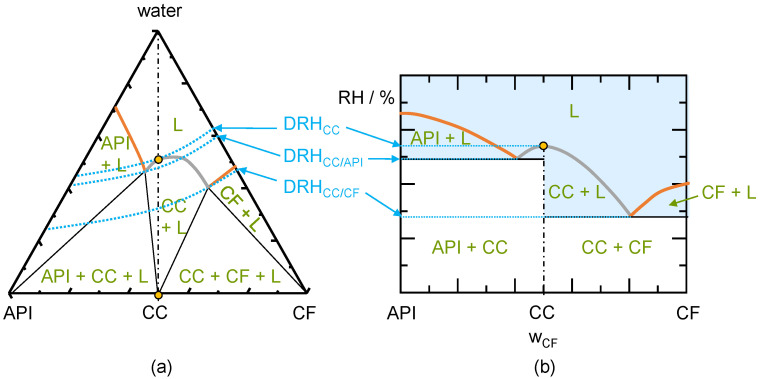
(**a**) Schematic ternary phase diagram for a congruently-dissolving CC. The three thick solid lines are the API solubility line, the CC solubility line, and the CF solubility line (from left to right). The thin solid lines indicate the phase boundaries, the thin dash-dotted line marks the CC stoichiometry. Dotted lines are iso-RH lines at DRH_CC_, DRH_CC/API_, and the DRH_CC/CF_; (**b**) Schematic phase diagram showing the thermodynamically-stable phases for a congruently-dissolving CC as function of the API/CF mass fraction in the dry system. Thick solid lines are deliquescence lines: API deliquescence, CC deliquescence line, and CF deliquescence line (from left to right). Thin solid lines indicate the phase boundaries, the thin dash-dotted line marks the CC stoichiometry. Dotted lines are the iso-RH lines at DRH_CC_, DRH_CC/API_, and the DRH_CC/CF_. The shaded region shows the presence of a liquid phase. The circles shown in (**a**,**b**) represent the equilibrium phases evolving at DRH_CC_.

The stability regions of a CC can be determined from phase diagrams. CC phase diagrams can be categorized into either congruently dissolving or incongruently dissolving CCs. Congruently dissolving CCs can establish a thermodynamically stable solution with stoichiometric composition. Contrary incongruently dissolving CCs cannot establish a thermodynamically stable solution with CC stoichiometric composition. The schematic phase diagrams for a congruently dissolving CC are shown in Figure 1. This system shows an almost symmetrical phase behavior (API solubility similar to CF solubility). The CC stoichiometry line crosses CC solubility line, which is the definition of a congruently dissolving CC. The API solubility line (separating region API and L), CC solubility line (separating region CC and L), and the CF solubility line (separating CF and L) separate the region of an unsaturated liquid phase (L) from the regions where crystals are present (below the solubility lines). Iso-RH lines are shown in the phase diagram to illustrate the influence of RH on the phase behavior. The RH (and therefore the water activity according to Equation (6)) along these lines is constant. DRH_CC_ is the RH above which a pure CC will start to deliquesce, and this value is determined by the water activity at the CC solubility line at CC stoichiometric composition (solid–liquid–vapor equilibrium). The DRH_CC/API_ is determined by the water activity at the eutectic point of the CC and the API (intersection of the CC solubility line and the API solubility line).

The DRH_CC/CF_ is determined by the water activity of the eutectic point of the CC and the CF (intersection of the CC solubility line and the CF solubility line). The iso-RH lines are only experimentally accessible in the liquid region (L) as both, vapor and liquid are required for the water to be in vapor–liquid equilibrium. Nevertheless, the iso-RH lines are also drawn in the metastable regions to improve their visibility. CC storage at DRH_CC_ will lead to the formation of a liquid phase with the composition on the solubility line (upper circle in Figure 1). This liquid phase is in equilibrium with the solid CC (lower circle in Figure 1a) and the vapor phase (indicated by the iso-RH line)

Figure 1b shows the influence of RH on the congruent phase behavior of the water-free system. The deliquescence lines separate the liquid phase (L) from regions, where crystals exist. As the transformation of a CC is assumed to occur through a liquid phase, it’s worth considering all regions with liquid phases (possibly besides crystals) [15,20]. Depending on the composition of the mixture, the liquid-phase formation (deliquescence) and therewith also the possible CC transformation occurs at different RH levels. Pure CC (on the CC stoichiometry line) is thermodynamically stable below DRH_CC_. A liquid phase will form next to the CC stored at the DRH_CC_ (compare circles in Figure 1a). This critical RH decreases when API crystals (even at trace levels) are present next to the CC. The composition moves slightly to the left of the CC stoichiometry and a liquid phase occurs at DRH_CC/API_. CF crystals next to the CC decrease this critical RH even further to DRH_CC/CF_ in this case. For low amounts of API or CF next to the CC, the CC will be present next to the liquid phase upon deliquescence (region CC + L). For higher amounts of API or CF next to the CC, either API or CF will coexist with the liquid phase and CC will completely dissolve in thermodynamic equilibrium (regions API + L or CF + L). If the RH is subsequently lowered below the critical RH values, the initially present crystals will crystallize again.

**Figure 2:**
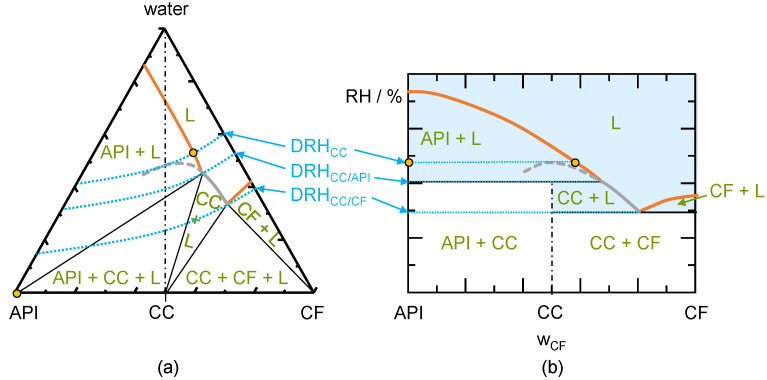
(**a**) Schematic ternary phase diagram for an incongruently-dissolving CC. The three thick solid lines are the API solubility line, the CC solubility line, and the CF solubility line (from left to right). The thick dashed line indicates the CC solubility line in the metastable region. The thin solid lines indicate the phase boundaries, the thin dash-dotted line marks the CC stoichiometry. Dotted lines are the iso-RH lines at DRH_CC_, DRH_CC/API_, and the DRH_CC/CF_; (**b**) Schematic phase diagram showing the thermodynamically-stable phases for an incongruently-dissolving CC as function of the API/CF mass fraction in the dry system. Thick solid lines are deliquescence lines: API deliquescence, CC deliquescence line, and CF deliquescence line (from left to right). The thick dashed line indicates the CC deliquescence line in the metastable region. The thin solid lines indicate the phase boundaries, the thin dash-dotted line marks the CC stoichiometry. Dotted lines are the iso-RH lines at DRH_CC_, DRH_CC/API_, and the DRH_CC/CF_. The shaded region shows the presence of a liquid phase. The circles shown in (**a**,**b**) represent the equilibrium phases evolving at DRH_CC_.

A schematic ternary phase diagram of an incongruently dissolving CC is shown in Figure 2a. Compared to congruently dissolving CC in Figure 1, the API solubility is significantly lower than the CF solubility. This leads to a shift of the CC region to the CF side of the diagram. Incongruently dissolving CCs cannot establish a thermodynamically stable solution with CC stoichiometric composition (CC stoichiometry line only crosses the metastable part of the CC solubility line). Therefore, the metastable CC solubility line is used to determine DRH_CC_. Analogously to the congruent phase behavior, DRH_CC_ is determined by the water activity on the CC solubility line at the stoichiometric composition. If an incongruently dissolving CC is stored at DRH_CC_, a liquid phase will evolve. This liquid phase (intersection of DRH_CC_ iso-RH line with the metastable CC solubility line) is supersaturated regarding the API in this exemplary case. Therefore, the API will crystallize and the liquid phase with the composition indicated as the circle on the API solubility line will evolve. In thermodynamic equilibrium, the CC is not stable and will be fully transformed into the API. Of course, the CC stored above DRH_CC_, might also transform to the CF or other polymorphs of the CC, the API, or the CF. These transformations depend on the inclination of the CC region in Figure 2a and on the kinetics of the crystallization. In this work, only the thermodynamically stable crystal form is evaluated.

Figure 2b shows a schematic phase diagram indicating the influence of RH on the dry mixture for an incongruently dissolving CC. The pure CC stored below the DRH_CC_ is thermodynamically stable although the phase diagram shows liquid-phase formation starting from DRH_CC/API_. These API and CF deliquescence lines are not valid for the pure CC but only the CC deliquescence line must be considered for the deliquescence. Thus, a liquid phase can only occur at storage above DRH_CC_. The liquid phase occurring above DRH_CC_ is supersaturated with respect to API (evolving liquid phase lies in the API + L region) and API will crystallize in thermodynamic equilibrium. The evolving phases in thermodynamic equilibrium at DRH_CC_ are indicated as circles in Figure 2b. When small amounts of API crystals are present next to the CC, a liquid phase can occur at DRH_CC/API_. The evolving liquid phase is again supersaturated with respect to API and API will crystallize. Thereby, CC crystals will transform into the API crystals in thermodynamic equilibrium. Small amounts of CF next to the CC will even lower the RH above which a liquid phase can occur to DRH_CC/CF_. Above this RH, the liquid phase is in equilibrium with the CC and therefore CF will completely dissolve (for compositions left of the eutectic composition of CC and CF). For compositions right of the eutectic point of CC and CF, CF will be stable above DRH_CC/CF_ and CC will completely dissolve.

To conclude, CCs with incongruent phase behavior in water can transform to either API or CF above their DRH_CC_ (depending on the location of the CC region). CC to API transformations are likely to occur with pharmaceutical CCs, since the API is the least water-soluble component of the CC. Congruent phase behavior may lead to unwanted CC dissolution above DRH_CC_ but as RH is decreased, CC is likely to crystallize again. This dissolution and subsequent recrystallization might alter the crystal surface area and therefore the performance of the CC. The knowledge of DRH_CC_, DRH_CC/API_, and DRH_CC/CF_ allows to determine the stability region for the CC depending on its purity and phase behavior (congruently dissolving or incongruently dissolving).

## 4. Materials and Methods

### 4.1. Materials

Carbamazepine (CBZ; form III; purity ≥ 98%), theophylline (TP; form II; purity of ≥99%), glutaric acid (GA; purity ≥ 99%), and the excipient xylitol (purity ≥ 99%) were obtained from Alfa Aesar (Karlsruhe Germany). Nicotinamide (NA; form I; purity ≥ 99.5%), citric acid (CA; purity ≥ 99.5%), the excipient D(-)fructose (purity ≥ 99%), and ethanol (purity ≥ 99.9%) were purchased from Sigma-Aldrich (Hamburg, Germany). Succinic acid (SA; purity ≥ 99.8%), urea (purity ≥ 99.5%), sodium chloride (NaCl, purity > 99%), potassium nitrate (KNO_3_, purity ≥ 99%), and methanol (purity ≥ 99.9%) were purchased from VWR Chemicals (Darmstadt, Germany). Potassium chloride (KCl, purity ≥ 99.5%) was purchased from Carl Roth (Karlsruhe, Germany) and sodium bromide (NaBr, purity > 99%) was purchased from Merck (Darmstadt, Germany). All chemicals were used without further purification. Water was deionized and filtered using a Merck Milli-Q Advantage A10 (Darmstadt, Germany) prior to use.

Several CC systems were investigated in this work. Congruent phase behavior is present for SA/NA (1:2) CC [26] and urea/GA (2:1) CC [27] in water, whereas urea/GA (1:1) CC [27] shows incongruent phase behavior in water. Moreover, two different CC stoichiometries can be observed for the urea/GA system [27]. The CBZ/NA/water system forms a 1:1 CC, while CBZ may also form a dihydrate (CBZ:water = 1:2) [28]. Hydrate formation of TP (1:1), CA (1:1) as well as of its CC (1:1:1) may occur for the TP/CA/water system [15].

The melting properties, the solubility products of the CCs and the properties used to calculate the hydrate solubility product are listed in Table 1, Table 2 and Table 3. The PC-SAFT pure-component parameters and the binary interaction parameters used to calculate the phase behavior are listed in Table 4 and Table 5.

### 4.2. Preparation of CCs

Defined compositions of API, CF, and solvent were weighed into a temperature-controlled 25 mL sealed glass vessel at 298 K (exact mass fractions are shown in the Supplementary Materials in Table S1). A magnetic stirrer was used to stir the suspension for at least 4 days to ensure thermodynamic equilibrium (preliminary tests showed that the thermodynamic equilibrium was reached within 4 days). Afterwards, the crystals were separated by a Büchner funnel and were dried in a second step under vacuum conditions for at least 7 days.

### 4.3. Stability Measurement

The prepared and dried CC samples were stored in sealed, temperature-controlled RH chambers at 298.15 K. A vacuum oven was used to keep 0% RH for the storage. Saturated salt solutions of sodium bromide (58% RH), sodium chloride (76% RH), potassium chloride (86% RH), potassium nitrate (93% RH), and potassium sulfate (98% RH) were prepared to obtain constant RH in each chamber [54]. The samples were investigated regarding their crystal form and gravimetrically before storage and after 1 day, 3 days, 7 days, 21 days, and 49 days. For that purpose, individual CC samples with an average of 40 mg (or 20 mg of CC and 10 mg of the excipient fructose or xylitol; see Tables S7–S9 in Supplementary Materials for exact mass fractions) were prepared beforehand in 2 mL Eppendorf reaction vessels for each investigated time and each storage condition. Thus, the influence of the subsequent investigations on samples was minimized.

The mass increase of each sample was measured gravimetrically. The water mass fractions for each investigated storage time are listed in the Supplementary Materials in Tables S2–S6. If the calculated mass fraction of water was below 0.02, it was assumed that deliquescence did not occur. Afterwards, each sample was analyzed by powder X-ray diffraction (PXRD) (Miniflex 600, Rigaku, Tokyo, Japan) with Cu Kα anode in reflection mode at a tube voltage of 40 kV and an electrical current of 15 mA. The diffractograms were collected at a scanning rate of 5° 2θ per minute from 5° to 35° 2θ. The storage conditions were not maintained during sample preparation and measurement in the PXRD. The crystal size was not investigated in this work, as only the thermodynamic equilibrium is evaluated. Of course, the crystal size influences the kinetics of the deliquescence and transformation but does not affect the thermodynamic equilibrium.

## 5. Results & Discussion

### 5.1. Influence of Congruency on Co-Crystal Stability

#### 5.1.1. Nicotinamide/Succinic Acid

**Figure 3:**
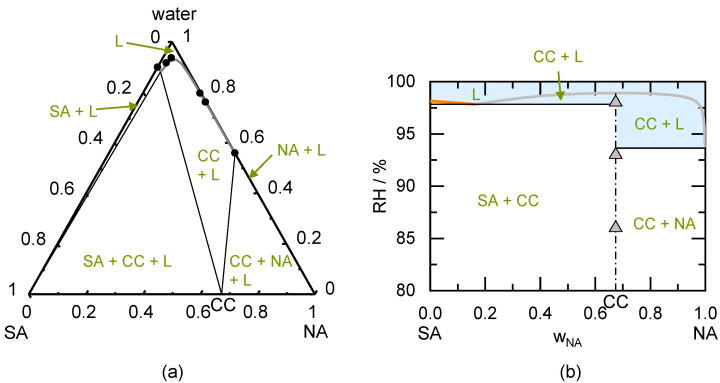
(**a**) Modeled ternary phase diagram of succinic acid/nicotinamide in mass fractions at 298.15 K. The SA solubility line starts from the left axis, the CC solubility line in the middle, and the NA solubility line starts from the right axis (cannot be seen in this diagram). The thin solid lines indicate the phase boundaries. The circles indicated solubility measurements obtained from the literature [26]; (**b**) Predicted phase diagram of succinic acid/nicotinamide showing the thermodynamically stable phases as function of the mass fraction in the dry system and RH at 298.15 K. The thick solid SA deliquescence line starts from the left axis intersecting the thick solid CC deliquescence line in the middle and the thick solid line of NA starts from the right axis (cannot be seen in this diagram). The thin solid lines indicate the phase boundaries, the thin dash-dotted line indicates the CC stoichiometry. Symbols indicate results of CC stability measurements after 49 days of storage. Triangles represent unchanged CC samples.

The PC-SAFT calculated ternary phase diagram of the NA/SA CC is shown in Figure 3a. Although the solubility of the SA is lower than the NA solubility, the CC is congruently dissolving. The experimentally determined solubility data were used to model the phase diagram [26].

Figure 3b shows the predicted phase regions depending on the storage RH. This phase diagram was predicted based on the simple solubility measurements shown in Figure 3a. According to the prediction, a physically pure SA/NA CC will be stable until 99% RH. When SA crystals are present next to the CC, liquid phase formation is favorable above 97.8% RH. This value is predicted to further decrease to 93.6% RH for the coexistence of NA crystals besides the CC. Due to its congruent phase behavior, the CC will not transform to SA nor NA between compositions of 0.16 < w_NA_ < 1 upon exceeding the DRH.

**Figure A1:**
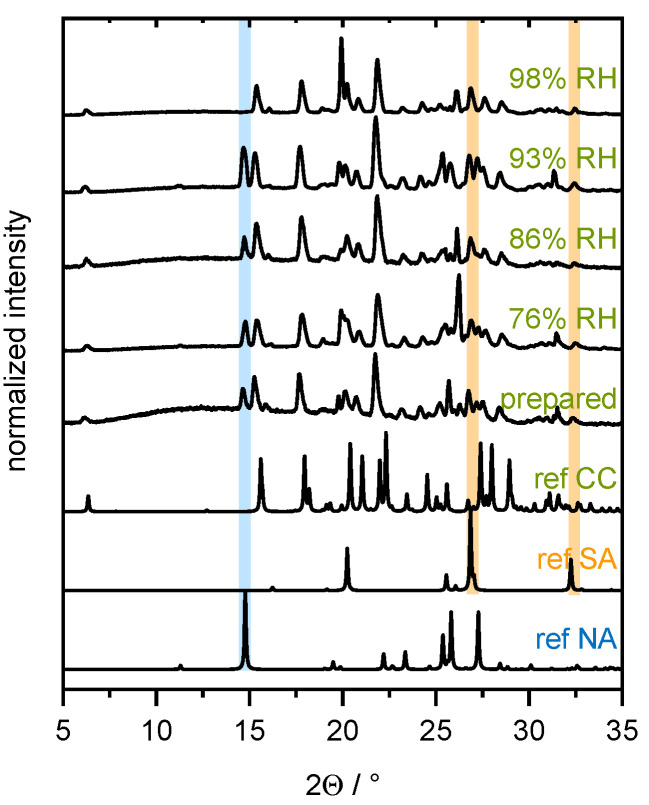
Measured diffractograms of stability measurements of the SA/NA CC. Reference (ref) diffractograms were obtained from the Cambridge Structural Database with following identifiers: CC (DUZPAQ), SA (SUCACB03), and NA (NICOAM). Highlighted peaks indicate the presence of the respective crystal next to the CC (NA and SA).

The PXRD analysis of the prepared CC sample showed SA crystals and NA crystals besides the CC (see PXRD diffractogram in Figure A1 in the Appendix A). Thus, the conversion of the prepared SA/NA mixture to the CC was not entirely completed during preparation. The peaks of SA crystals and NA crystals remained present during storage of the samples below 98% RH. Only at 98% RH, the peaks disappeared (probably due to deliquescence and subsequent CC crystallization from the solution). The predicted DRH_SA/NA_ which must be reached to allow CC formation is 90.1%. Storage at 93% RH for 49 days did not lead to the conversion to CC but NA and SA crystals remained present.

Table 6 shows the results of the CC stability measurements after 49 days (longest investigated period). Deliquescence occurred only at higher RH (above DRH) and all samples remained unchanged below DRH. All investigated samples were predicted to deliquesce at 98% RH and the addition of excipient sugars decreased the DRH compared to the excipient-free systems. The predicted range for DRH very well agrees to the experimentally observed deliquescence.

The prepared SA/NA samples stored at the investigated RH conditions (0% RH to 98% RH) did not absorb more than 2% water within 49 days of storage (were assumed to not deliquesce). Nevertheless, it was predicted that deliquescence occurs above 97.8% RH (due to the presence of SA crystals besides CCs). The small driving force resulting from the difference in storage RH to DRH might have prevented significant water sorption within 49 days of storage at 98% RH. In agreement to the prediction, the prepared congruently dissolving CCs did not convert during storage at all investigated RH conditions.

#### 5.1.2. Urea/Glutaric Acid

**Figure 4:**
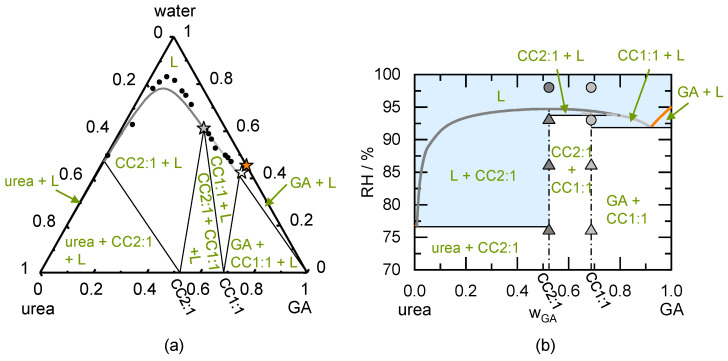
(**a**) Modeled ternary phase diagram of urea/glutaric acid in mass fractions at 298.15 K. The urea solubility line starting from the left axis, the CC2:1 solubility line in the middle intersecting into the CC1:1 solubility line. The GA solubility line starts from the right axis (not to be seen). The thin solid lines indicate the phase boundaries. The circles indicated solubility measurements obtained from the literature [27]. The stars indicate measurements used to fit the binary interaction parameters and solubility products of the CCs; (**b**) Predicted phase diagram of urea/glutaric acid showing the thermodynamically stable phases as function of the mass fraction in the dry system and RH at 298.15 K. The thick solid urea deliquescence line starting from the left axis immediately intersecting the thick solid CC2:1 deliquescence line in the middle, which is intersecting into the CC1:1 deliquescence line. The thick solid deliquescence line of GA starts from the right axis. The thin solid lines indicate the phase boundaries, the thin dash-dotted lines indicate the CC stoichiometries. Dark grey symbols indicate results of CC2:1 stability measurements after 49 days of storage and light grey symbols indicate results of CC1:1 stability measurements after 49 days of storage. Triangles represent unchanged CC samples. Circles indicates deliquescence during storage.

Another example for a congruently dissolving CC (in water) is the urea/GA CC2:1. The modeled ternary phase diagram can be seen in Figure 4a. According to the phase diagram, urea/GA may form two CCs of different stoichiometry. The CC2:1 is congruently dissolving and the CC1:1 is incongruently dissolving in water. The measured aqueous solubility of GA is only slightly higher than the aqueous solubility of urea [27]. The phase behavior was modeled using three experimentally determined solubilities (shown as stars in Figure 4a) [27]. The GA aqueous solubility was used to determine the binary interaction parameter between water and GA. The binary interaction parameter between urea and GA was determined by fitting the GA aqueous solubility in presence of urea to the measured eutectic point between GA and CC1:1 (white star in Figure 4a). The solubility products of CC2:1 and CC1:1 were determined using the measured aqueous solubility at the eutectic point of CC2:1 and CC1:1. The modeled phase diagram slightly deviates from the measured urea/GA CC2:1 solubility, while it agrees well with the measured solubility data of urea/GA 1:1 CC obtained from the literature [27].

The phase diagram Figure 4a was used to predict the phase diagram showing the RH influence on the dry system in Figure 4b. The DRH_urea_ (76.7% RH) is significantly lower than the DRH_GA_ (95% RH), although the aqueous solubility might suggest an opposite behavior (higher solubility is usually connected to a lower DRH [55]).

Pure CC2:1 is predicted to be stable until 94.7% RH (DRH_CC2:1_). The DRH of CC2:1 significantly decreases (to 76.6% RH) in the presence of urea crystals next to the CC2:1. If GA crystals coexists with CC2:1, DRH_CC2:1/GA_ is predicted to be 91.4% RH (not shown in the phase diagram). DRH_CC1:1_ is 94% RH, in presence of the urea DRH_CC1:1/urea_ is 75.9% RH, and in presence of GA, DRH_CC1:1/GA_ is 91.9% RH (all values predicted). Above these values, the samples will start to deliquesce. According to the prediction, CC2:1 will not transform to any other form upon deliquescence and subsequent drying (congruent phase behavior). Contrary, CC1:1 is predicted to transform into CC2:1 between 93.7% RH and 94% RH (region CC2:1 + L). Storage above this RH leads to a full CC dissolution.

**Figure A2:**
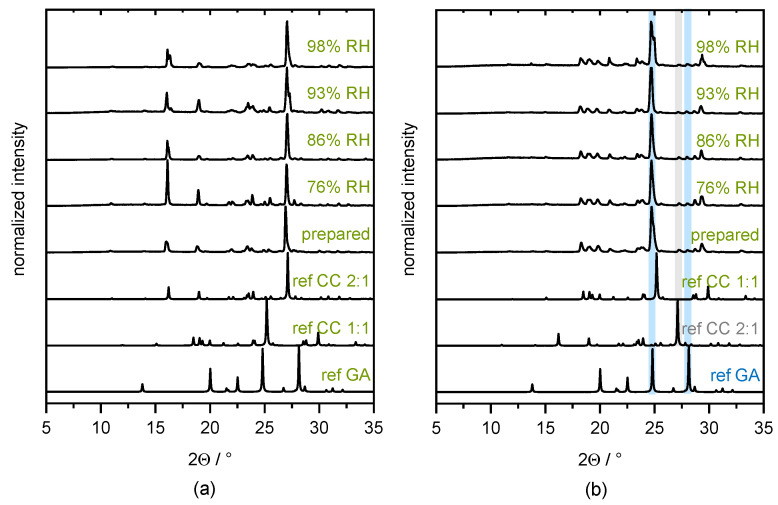
Measured diffractograms of stability measurements of the (**a**) urea/GA CC2:1 and (**b**) urea/GA CC1:1. Reference (ref) diffractograms were obtained from the Cambridge Structural Database with following identifiers: CC 2:1 (TONGOS), CC 1:1 (ZODWIY01), and GA (GLURAC). Highlighted peaks indicate the presence of the respective crystal next to the CC1:1 (CC2:1 and GA).

The diffractogram revealed that the prepared CC2:1 is pure (according to the PXRD), whereas the prepared CC1:1 additionally contained CC2:1 and GA crystals (compare Figure A2 in the Appendix A). CC2:1-deliquescence was -in agreement to the prediction- only observed at 98% RH. CC1:1 deliquesced -in agreement to the prediction- also at 93% RH, because it coexisted with GA (DRH_CC1:1/GA_ = 91.9% RH). Thus, the predicted DRH values were in excellent agreement with observations from the stability measurements. Neither a transformation of CC1:1 nor crystallization of CC2:1 did occur within 49 days of storage at the here-investigated conditions (compare also Table 6). Nevertheless, according to the predicted phase diagrams, there exists a small RH range (93.7% to 94% RH), in which CC1:1 would transform to CC2:1. The investigated RH conditions did not lie within this range and therefore the transformation was not observed experimentally.

### 5.2. Influence of API Hydrate Formation on Co-Crystal Stability

#### Carbamazepine/Nicotinamide

**Figure 5:**
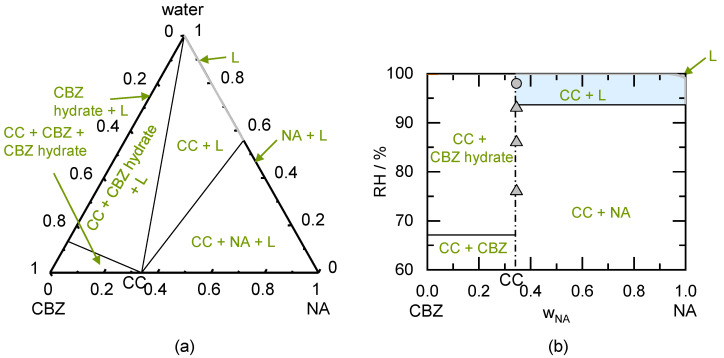
(**a**) Predicted ternary phase diagram of carbamazepine/nicotinamide in mass fractions at 298.15 K. The CBZ hydrate solubility line starting from the left axis, the CC solubility line in the middle, and the NA solubility line starting from the right axis (lines are very close to the axis). The thin solid lines indicate the phase boundaries; (**b**) Predicted phase diagram of carbamazepine/nicotinamide showing the thermodynamically stable phases as function of the mass fraction in the dry system and RH at 298.15 K. The thick solid CBZ hydrate deliquescence line starting from the left axis intersecting the thick solid CC deliquescence line in the middle and the thick solid line of NA starts from the right axis (lines are very close to the axis). The thin solid lines indicate the phase boundaries, the thin dash-dotted line indicates the CC stoichiometry. Symbols indicate results of CC stability measurements after 49 days of storage. Triangles represent unchanged CC samples. The circle indicates deliquescence during storage.

CBZ forms a dihydrate above 65% RH at 298.15 K [56,57]. The influence of this hydrate formation on the stability of the CBZ/NA CC was investigated based on the ternary phase diagram of CBZ/NA/water shown in Figure 5a. The solubility of NA and the solubility of CBZ hydrate in water were previously modeled [19,26] and the CC aqueous solubility was predicted on the basis of a modeled CBZ/NA/methanol phase diagram [18]. The solubility of the CBZ hydrate is by magnitudes lower than that of NA. Nevertheless, the resulting CC is congruently dissolving. The influence of RH on the dry system shown in the predicted phase diagram in Figure 5b indicates that the values for DRH_CBZ hydrate_, DRH_CC_, as well as DRH_CC/CBZ hydrate_ are close to 100% RH. Therefore, the CC (even in the presence of CBZ hydrate crystals) is predicted to be stable up to almost 100% RH. Only the presence of NA crystals next to the CC allows for deliquescence at a lower predicted DRH_CC/NA_ of 93.6% RH. Nevertheless, the storage of this CC in presence of NA will only lead to the dissolution of NA, but CC will still remain present up to 100% RH according to the prediction (region CC + L).

**Figure A3:**
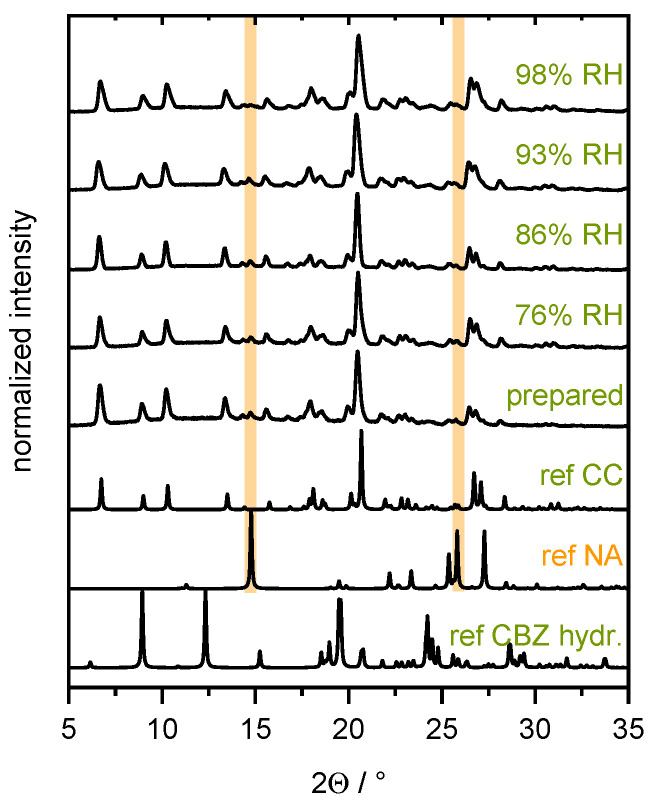
Measured diffractograms of stability measurements of the CBZ/NA CC. Reference (ref) diffractograms were obtained from the Cambridge Structural Database with following identifiers: CC (UNEZES), NA (NICOAM), and CBZ dihydrate (FEFNOT). Highlighted peaks indicate the presence of NA next to the CC.

The prepared sample for the stability measurement was investigated using PXRD. The diffractogram shown in Figure A3 in the Appendix A indicates that crystalline NA coexisted besides the CC. Therefore, deliquescence could have been occurred already above the predicted DRH_CC/NA_ of 93.6% RH. Indeed, deliquescence only occurred during storage at 98% RH with an absorbed water mass fraction of 0.022 (compare Table 6).

According to the predicted phase diagram and previous investigations [19], the CBZ anhydrate crystal (form III) is only stable below 67% RH at 298.15 K (agrees with the measured value of 65% RH at 298.15 K [56]). Above this RH, CBZ form III transforms to the CBZ dihydrate with different physicochemical properties compared to the CBZ form III. This abrupt change in physicochemical properties was prevented by formulating CBZ as a CBZ/NA CC. Thus, the critical hydrate formation RH of CBZ did not affect the physically pure CC, which was predicted to be stable up to 100% RH. Although the presence of NA crystals next to the CC lowered this value to 93.6% RH, the hydrate formation capability of CBZ did not affect the stability of the CC and thus a transformation of CBZ into the CBZ hydrate could be prevented.

Our predicted phase diagrams also explain the formation of the CBZ/NA CC by moisture sorption of a mixture of CC components in the presence and absence of sugar additives reported by Jayasankar et al. [13]. CBZ/NA CC formation was observed in the absence of sugar additives at 98% RH, which agrees with the predicted DRH_CBZ/NA_ of 93.5% (not shown in the phase diagram in Figure 5) and the findings in Table 6. Furthermore, the reported CC formation in presence of fructose at 75% and 85% RH is in agreement with the predicted deliquescence in Table 6 (predicted DRH_CBZ/NA/fructose_ is 49%; not shown in Table 6).

### 5.3. Influence of Co-Crystal Hydrate Formation on Co-Crystal Stability

#### Theophylline/Citric Acid

**Figure 6:**
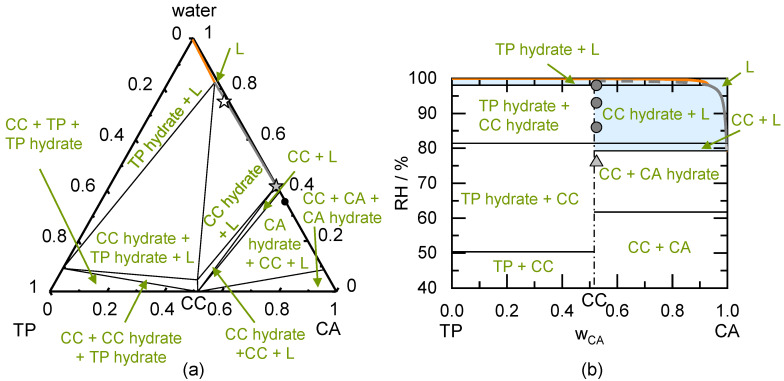
(**a**) Modeled ternary phase diagram of theophylline/citric acid in mass fractions at 298.15 K. The TP hydrate solubility line starting from the left axis, the CC hydrate solubility line below is intersecting into the CC solubility line. The CA hydrate solubility line is starting from the right axis (cannot be seen in this diagram). The thin solid lines indicate the phase boundaries. The circle indicates the eutectic point of CA hydrate and the CC anhydrate, the grey star is the eutectic point of the CC hydrate and CC anhydrate, and the white star is the eutectic point of the CC hydrate and TP hydrate obtained from literature [15]; (**b**) Predicted phase diagram of theophylline/citric acid showing the thermodynamically stable phases as function of the mass fraction in the dry system and RH at 298.15 K. The thick solid TP hydrate deliquescence line starting from the left axis intersecting the thick solid CC hydrate deliquescence line, which is intersecting in the CC anhydrate deliquescence line. The thick solid line of CA hydrate starts from the right axis (cannot be seen in this diagram). The thick dark-grey dashed line indicates the metastable CC hydrate deliquescence line. The thin solid lines indicate the phase boundaries, the thin dash-dotted line indicates the CC stoichiometry. Symbols indicate results of CC hydrate stability measurements after 49 days of storage. The light grey triangle represent transformation to the CC anhydrate. Dark grey circles indicate deliquescence during storage of CC hydrate.

TP and CA can form a CC anhydrate or a CC hydrate in presence of water [15]. Thus, it is of particular interest to know the stability regions of the CC anhydrate or the CC hydrate. TP and CA each may also form hydrates, which was previously investigated [16,19]. The modeled ternary phase diagram is shown in Figure 6a. The binary interaction parameter between TP and CA was fitted to the solubility of TP hydrate in presence of CA at the eutectic point between TP hydrate and the CC hydrate (white star in Figure 6a). The solubility product of the CC anhydrate and CC hydrate was fitted to the solubility at the eutectic point of CC anhydrate and CC hydrate (grey star in Figure 6a). The modeled ternary phase diagram indicates that the solubility of TP hydrate is magnitudes lower than the solubility of CA hydrate. This results in an incongruently dissolving CC anhydrate as well as an incongruently dissolving CC hydrate in water.

The RH influence on the dry system is indicated in the predicted phase diagram shown in Figure 6b. The diagram is separated into different stability regions depending on the RH. According to the prediction, the CC anhydrate is stable below 81.5% RH at 298.15 K and will transform to the CC hydrate above 81.5% RH. This is in good agreement with the experimentally determined critical RH for hydrate formation found in the literature of 80% RH [15]. For an RH above 99.3% RH (DRH_CC hydrate_), CC hydrate will deliquesce and transform to the TP hydrate (DRH_TP hydrate_ ≈ 100% RH). Left of the CC stoichiometry (excess of TP crystals next to the CC) and below 50.4% RH, CC anhydrate is stable besides TP anhydrate. Above 50.4% RH and below 81.5% RH, TP anhydrate transforms to the TP hydrate in presence of the CC anhydrate. The CC anhydrate transformation to CC hydrate in presence of TP hydrate is predicted to be above 81.5% RH and below 98.1% RH. Only above 98.1% RH (DRH_CC hydrate/TP hydrate_), deliquescence occurs, and the CC hydrate will dissolve and only TP hydrate will be stable in thermodynamic equilibrium (region TP hydrate + L).

To the right of the CC stoichiometry (excess of CA crystals next to the CC), the CC anhydrate coexists besides the CA anhydrate below 61.8% RH. CA anhydrate transforms to CA hydrate in presence of CC anhydrate above 61.8% RH and below 79.3% RH. Deliquescence occurs above 79.3% RH (DRH_CC/CA hydrate_) and CA hydrate dissolves besides the CC anhydrate. Again, above 81.5% RH the CC anhydrate transforms to CC hydrate, this time, in presence of a liquid phase. A transformation of CC hydrate to TP hydrate will occur above 98.1% RH for a CA mass fraction below eutectic concentrations of TP hydrate and CC hydrate. For a CA mass fraction higher than this, all crystals will dissolve into an unsaturated liquid phase above 98.1% RH.

**Figure A4:**
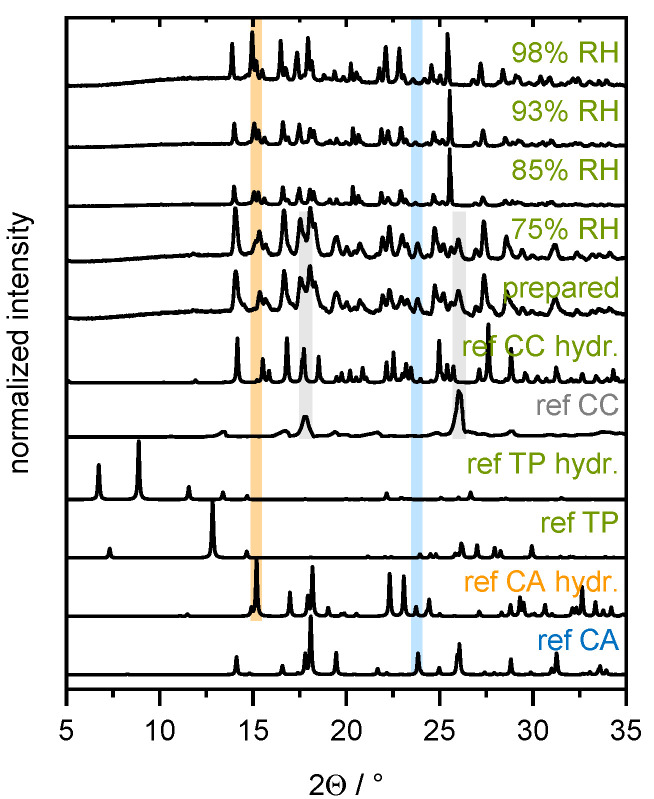
Measured diffractograms of stability measurements of the TP/CA CC hydrate. Reference (ref) diffractograms were obtained from the Cambridge Structural Database with following identifiers: CC hydrate (KIGKAN), CC anhydrate (extracted from Jayasankar [59]), TP hydrate (THEOPH), TP anhydrate (BAPLOT07), CA hydrate (CITARC), and CA anhydrate (CITRAC10). Highlighted peaks indicate the presence of the respective crystal next to the CC hydrate (CC anhydrate and CA hydrate).

Due to the very small CC + L region in the phase diagram of TP/CA/water (compare Figure 6a), a mixture with a composition in the CC hydrate + L region was prepared to form the CC hydrate. The analysis of the prepared sample after drying showed that next to CC hydrate, CC anhydrate, and CA hydrate were present (compare Figure A4 in the Appendix A). The presence of CC anhydrate is not surprising, because the sample was stored under vacuum conditions for at least seven days. CC hydrate is not stable under vacuum conditions due to the absence of water and being far below the critical hydrate formation RH (80% RH [15]). Due to the presence of CA anhydrate or CA hydrate next to the CC hydrate, the prepared sample can be found slightly right of the CC stoichiometric line in Figure 6b. Deliquescence was observed -in agreement to the prediction- for samples stored at 86% RH, 93% RH, and 98% RH (compare Table 6). At 76% RH, the sample did not deliquesce but transform to CC anhydrate (compare Figure A4 in the Appendix A). This agrees with the predicted critical CC hydrate formation RH of 81.5% RH. The CA hydrate peak at 24° 2 θ can clearly be seen in the prepared sample and at 76% RH, but this peak disappears from the diffractograms for samples stored at 86% RH and above. This agrees to the prediction as CA hydrate should deliquesce above 79.3% RH. CC hydrate remained unchanged (after dissolution and subsequent crystallization upon drying) at 86% RH, 93% RH, and 98% RH storage. Nevertheless, CC hydrate is predicted to transform to TP hydrate above 98.1% RH (not investigated in this work).

Jayasankar et al. [15] investigated the transformation pathways of a TP/CA physical mixture exposed to 75% RH, 85% RH, and 98% RH. In agreement with the predicted DRH_TP/CA_ of 74.9% RH (not shown in Figure 6b), a liquid phase evolved at all investigated storage conditions. CC anhydrate initially crystallized from the liquid phase at all investigated storage conditions. CC anhydrate was stable at 75% RH, transformed to CC hydrate at 85% RH, and transformed to CC hydrate as well as TP hydrate at 98% RH within 4 weeks. This confirms the predicted phase diagram in Figure 6b. TP hydrate formation was predicted to occur above 98.1% RH, which is close to the storage condition of 98% RH. The fact that TP hydrate occurred within four weeks of storage at 98% RH (Jayasankar et al. [15]), but did not occur within seven weeks of our investigation, might result from the different starting conditions of the experiment (TP/CA physical mixture for Jayasankar et al. [15] and the CC hydrate besides CA hydrate for our investigation). The presence of even trace-level amounts of crystalline TP might decrease the kinetic barrier for TP hydrate crystallization.

To conclude, the CC hydrate-formation capability influences the CC stability. A certain critical hydrate formation RH determines whether the CC anhydrate or the CC hydrate is stable. This critical RH only depends on temperature [19,47]. The hydrate formation capability of the API (TP in this case) or CF (CA in this case) does not influence the CC stability. Solely, the presence of either API or CF (also as hydrates) besides the CC might drastically decrease DRH_CC/API_ or DRH_CC/CF_ relative to the DRH_CC_. In those cases, deliquescence and CC transformation into other crystal forms might occur at lower RH compared to pure CC.

### 5.4. Influence of Excipients on Co-Crystal Stability

**Figure 7:**
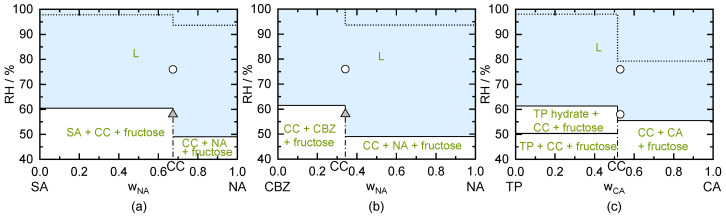
Predicted phase diagrams of (**a**) SA/NA, (**b**) CBZ/NA, and (**c**) TP/CA as function of the mass fraction of the dry, fructose-free system and of RH in the presence of fructose next to the CC at 298.15 K. Thin solid lines indicate phase boundaries. Vertical dash-dotted lines mark CC stoichiometries. Above the solid horizontal lines (shaded region L), deliquescence is predicted to occur in case that fructose is present, whereas as above the dotted lines, deliquescence is predicted to occur in the fructose-free system. Symbols indicate results of CC/fructose stability measurements after 49 days of storage. Triangles represent unchanged samples. White circles indicate occurrence of deliquescence during storage.

**Figure 8:**
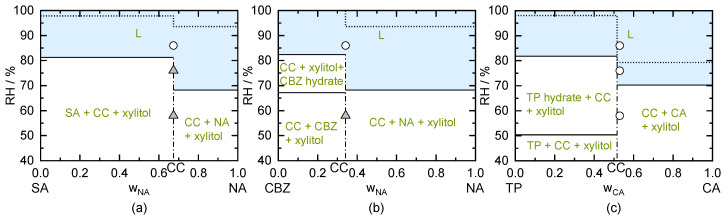
Predicted phase diagrams of (**a**) SA/NA, (**b**) CBZ/NA, and (**c**) TP/CA showing the thermodynamically stable phases depending on the mass fraction of the dry, xylitol-free system and RH for the presence of xylitol next to the CC at 298.15 K. Thin solid lines indicate phase boundaries. Vertical dash-dotted lines mark CC stoichiometries. Above the solid horizontal lines (shaded region L), deliquescence is predicted to occur in case that xylitol is present, whereas as above the dotted lines, deliquescence is predicted to occur in the xylitol-free system. Symbols indicate results of CC/xylitol stability measurements after 49 days of storage. Triangles represent unchanged samples. White circles indicate the occurrence of deliquescence during storage.

It was shown that the presence of even trace levels of API or CF (or their hydrates) besides the CC (or CC hydrate) drastically reduces the CC stability due to deliquescence at lower DRH. The influence of additional excipient crystals besides the CC was investigated for the example excipients fructose and xylitol. The excipients were selected due to their low DRH value to show the considerable influence the excipients can have on the CC stability. The excipient influence on the stability of the before-investigated CCs (Section 5.1, Section 5.2 and Section 5.3) is shown in predicted phase diagrams in Figure 7 and Figure 8. These phase diagrams again show the regions of deliquescence (region indicated with L). The composition of the locally evolving liquid phase (eutectic composition at the DRH_CC/excipient_) might be different from the bulk composition of the prepared sample. Therefore, the regions for deliquescence are independent of the overall CC/excipient mass ratio present in the prepared sample. Next to CC and excipient crystals, API and CF crystals (or their hydrates) again influence the DRH value of the crystal mixture. Regions of deliquescence were predicted based on the DRH of those crystal mixtures which are critical to the stability of the prepared CC/excipient mixture. The binary interaction parameters between the excipients and water were obtained from literature [52]. Binary interaction parameters between the excipients and every other component were set to zero.

#### 5.4.1. Fructose

The influences of fructose on the SA/NA CC, the CBZ/NA CC, and the TP/CA CC hydrate are indicated in Figure 7. Fructose has a predicted DRH_fructose_ of 61.5% RH, which perfectly agrees with the DRH measurement of 62% RH [58]. Thus, for any CC, fructose as excipient will lead to deliquescence values below this value (predicted DRH_CC/fructose_ of 60.5% of SA/NA CC and 61.5% of CBZ/NA CC as well as pure TP/CA CC). By comparing the fructose-free system (indicated as dotted lines in Figure 7) with the system including fructose (solid lines), it becomes obvious that fructose should not be used as an excipient for the formulation of any of these CCs. If the CC coexists with fructose and NA crystals, the predicted DRH_CC/NA/fructose_ of the systems SA/NA and CBZ/NA is as low as 49% RH (see Figure 7a,b). If TP/CA CC hydrate coexists with fructose and CA crystals, the DRH_CC hydrate/CA/fructose_ was predicted to be 55.6% RH. In such a formulation (mixture of CC hydrate, CA, and fructose), even moderate storage RH conditions might thus lead to CC deliquescence.

To verify the predictions, stability measurements of CC/fructose mixtures were performed at 58% RH and 76% RH. The prepared SA/NA CC in presence of fructose (see Figure 7a) did not show deliquescence at 58% RH but deliquesces at 76% RH in agreement to the prediction (compare Table 6). Due to the presence of SA crystals and NA crystals (compare Section 5.1), slight deliquescence might have happened between the SA crystals and NA crystals (predicted DRH_SA/NA/fructose_ = 49% RH) at 58% RH. Nevertheless, the evolving liquid phase is supersaturated with respect to CC and CC will crystallize when stored above 49% RH. According to the diffractograms at 58% RH and 76% RH, NA seems to crystallize during storage at 76% RH as the thermodynamically stable form. Thus, not only dissolution of the CC occurred at 76% RH, but a transformation into a thermodynamically stable form (NA in this case) happened.

**Figure A5:**
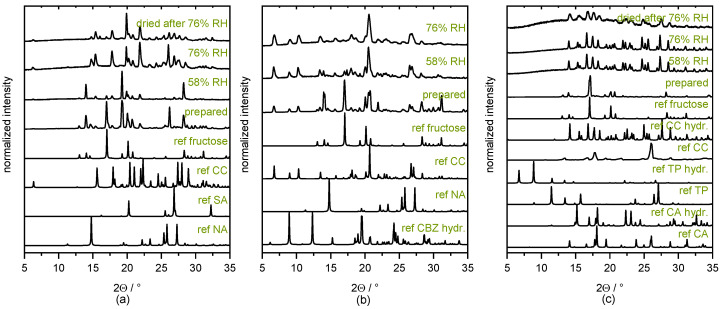
Measured diffractograms of stability measurements of the CC in presence of fructose: (**a**) SA/NA CC; (**b**) CBZ/NA CC; (**c**) TP/CA CC hydrate. Reference (ref) diffractograms were obtained from the Cambridge Structural Database with following identifiers: SA/NA CC (DUZPAQ), SA (SUCACB03), NA (NICOAM), CBZ/NA CC (UNEZES), CBZ dihydrate (FEFNOT), TP/CA CC hydrate (KIGKAN), TP/CA CC anhydrate (extracted from Jayasankar [59]), TP hydrate (THEOPH), TP anhydrate (BAPLOT07), CA hydrate (CITARC), and CA anhydrate (CITRAC10).

The sample of CBZ/NA CC together with fructose crystals (see Figure 7b) was unchanged at 58% RH but deliquesced at 76% RH (compare Table 6). Therefore, the measurement agrees to the predicted DRH value for this system (61.5% RH). A transformation of the CC into another crystal was not observed. Storage of TP/CA CC hydrate besides fructose (see Figure 7c) crystals led to deliquescence at 58% RH as well as at 76% RH (compare Table 6). According to the diffractograms (Figure A5c in the Appendix A), CA was present next to the TP/CA hydrate and fructose. Therefore, the sample is predicted to deliquesce above the DRH_CC hydrate/CA/fructose_ of 55.6% RH, which agrees with the findings.

According to the prediction, CC hydrate should transform to CC anhydrate in thermodynamic equilibrium (CC hydrate is predicted to be not stable below 81.5% RH at 298.15 K). According to the diffractograms (Figure A5c in the Appendix A), the conversion to CC anhydrate did not occur within 49 days of storage. The liquid phase might have been too viscous to allow a transformation within 49 days. Still, the transformation is expected to occur in thermodynamic equilibrium.

#### 5.4.2. Xylitol

The predicted phase diagrams for storing SA/NA CC, CBZ/NA CC, and TP/CA CC hydrate each in presence of xylitol crystals are shown in Figure 8. Pure xylitol has a predicted DRH_xylitol_ of 82.4% RH which reasonably agrees with the measured DRH of 79% from the literature [58]. Pure SA/NA CC in the presence of xylitol is predicted to deliquesce above 81.4% RH. CBZ/NA CC as well as the TP/CA CC hydrate, both in the presence of xylitol, were predicted to deliquesce at 82.4% RH. An addition of NA crystals to SA/NA CC or to CBZ/NA CC, both in the presence of xylitol crystals, decreases the predicted DRH value to 68.2% RH (compare Figure 8a,b). An addition of CA crystals to TP/CA CC hydrate in the presence of xylitol crystals lowers the predicted DRH to 69%. Compared to the DRH limit above which deliquescence occurs in the xylitol-free system (dotted line in Figure 8), deliquescence will again occur at significantly lower RH values in the presence of xylitol crystals.

The stability measurements of the CC in presence of xylitol crystals were performed at 58% RH, 76% RH, and 86% RH (CBZ/NA CC was not stored at 76% RH in this investigation). In agreement to the prediction, SA/NA CCs in the presence of xylitol crystals (see Figure 8a) did not deliquesce at 58% RH and 76% RH but deliquesces at 86% RH (compare Table 6). CBZ/NA CC in presence of xylitol crystals (see Figure 8b) did not deliquesce at 58% RH, whereas storage at 86% RH led to deliquescence (compare Table 6). This was expected according to the prediction (predicted DRH_CC/xylitol_ of 82.4%).

The stability measurement of TP/CA CC hydrate with xylitol crystals (see Figure 8c) showed deliquescence for all storage conditions, although deliquescence was not predicted for 58% RH (compare Table 6). The interactions between xylitol and CA seem to be underestimated by the model and therefore, the predicted DRH is higher than the experimental one (predicted DRH_CC/xylitol/CA_ is 69%). TP/CA CC hydrate was expected to transform to the CC anhydrate during storage at 58% RH and 76% RH (below predicted critical hydrate formation RH of 81.5% RH at 298.15 K). Nevertheless, CC hydrate remained stable for 49 days of storage, even though the CC anhydrate is thermodynamically stable below 80% RH [15]. Even drying of the sample stored at 86% RH for another 49 days under vacuum conditions did not lead to the transformation of CC hydrate to CC anhydrate. Thus, the presence of xylitol significantly decreased the dehydration kinetics.

To conclude, the use of additional excipients crystals for any of the here-investigated CCs decreased the DRH significantly. As expected, crystalline excipients with low DRH result in deliquescence at lower RH for the crystal mixture of CC/excipient. Upon deliquescence, the evolving liquid phase also partly dissolves the CC and thus allows for a transformation of the CC into another thermodynamically more-stable crystal form (see SA/NA CC transforming to NA crystals at 76% RH in the presence of fructose). In contrast, it was observed that the transformation of the TP/CA CC hydrate into the TP/CA CC anhydrate could be prevented for 49 days of storage below the critical CC hydrate formation RH in presence of the excipients.

## 6. Conclusions

Phase diagrams (crystal solubility, deliquescence relative humidity (DRH)) predicted via PC-SAFT allow to determine the critical storage conditions at which CCs become unstable. The phase diagrams can be predicted only based on simple solubility measurements of the CC in water or any other solvent. The stability of a CC against RH depends on three influencing factors:

First, if the CC might form CC hydrates, the critical CC-hydrate RH needs to be considered. The CC anhydrate is stable below the critical RH of the CC hydrate, whereas the CC hydrate forms above this RH.

The second factor is the DRH of the CC, which significantly depends on the presence of the crystal form (CC anhydrate or CC hydrate) and on the presence of other coexisting crystal forms (active pharmaceutical ingredient (API), coformer (CF), or excipient crystals). The investigations showed that even small excess amounts of API crystals or CF crystals next to the CC significantly lower the DRH and therewith decrease the stability of the CC. Contrary, formation of API hydrates or CF hydrates does not affect the CC stability aside from the DRH decrease. In fact, hydrate formation of either API or CF can be even prevented by formulating the API as a CC. Sugar excipients considered in this work were predicted to significantly decrease the DRH. The DRH of the pure excipient crystal is a suitable indicator for the DRH decrease in the CC/excipient system. A liquid phase forms (deliquescence) for RHs above the DRH of the crystal mixture, which is a prerequisite for the unwanted transformation of the CC into any other stable crystal form. This transformation is only occurring if the CC is incongruently dissolving.

That’s why the third factor is the congruence of the CC/water phase diagram. For congruently dissolving CCs, deliquescence is reversible, and the CC crystallizes again when RH is decreased below DRH. Incongruently dissolving CCs might irreversibly transform to another crystal form upon deliquescence. All predictions were validated by stability measurements of CCs (with excess API/CF or additional excipients). The PC-SAFT predictions were in excellent agreement with the CC stability measurements at humid conditions. Thus, this approach is considered as being able to predict the stability of any CC in combination with any crystalline excipient.

## Figures and Tables

**Table 1:** Melting properties of the components investigated in this work.

Component	$T_{0i}^{SL}/K$	$\Delta h_{0i}^{SL}/\mathrm{kJ}\;{\mathrm{mol}}^{-1}$	$\Delta c_{p,0i}^{SL}/J\;{\mathrm{mol}}^{-1}\;K^{-1}$	References
CA	428.55	41.84	159.46 ^a^	[29,30]
CBZ form III	447.95	26.81	65.17	[31,32]
GA	370.95	20.9	107.45	[33,34,35]
NA form I	401.15	28.0	78.12	[36,37]
SA	461.15	38.91	69.79	[38]
TP form II	542.25	28.2	114.91	[33,39,40]
urea	405.8	13.61	0	[41]
fructose	353.15	32.4	0	[42]
xylitol	363.15	38.0	0	[42]

^a^ It was assumed that the heat capacity of the glassy state is similar to the heat capacity of the solid state [43].

**Table 2:** Solubility products of the investigated CCs at 298.15 K.

CC	Stoichiometry	$K_{s,CC}\;*$	Reference for Properties	Reference for Experimental Data
CBZ/NA	1:1	7.26 × 10^−8^	[18]	[28]
NA/SA	2:1	3.70 × 10^−7^	[26]	[26]
TP/CA	1:1	6.09 × 10^−5^	this work	[15]
TP/CA/water	1:1:1	4.95 × 10^−5^	this work	[15]
urea/GA	2:1	2.00 × 10^−4^	this work	[27]
urea/GA	1:1	6.37 × 10^−3^	this work	[27]

* $K_{s,\mathrm{CC}}$ is without unit since it is based on thermodynamic activities.

**Table 3:** Properties used to calculate the hydrate solubility product and the resulting hydrate solubility product at 298.15 K.

Hydrate	Stoichiometry	Transition Polymorph	$T^{trans}/K$	$\Delta h^{trans}/\mathrm{kJ}\;{\mathrm{mol}}^{-1}$	$K_{s,hydrate}\;*\;\mathrm{at}\;298.15\;K$	References
CA/water	1:1	anhydrate	309.45	10.85	0.0152	[16,44,45]
CBZ/water	1:2	III	343.15	15.06	0.0256	[19,46]
TP/water	1:1	II	340	13.81	0.0634	[19,47]

* $K_{s,\mathrm{hydrate}}$ is without unit since it is based on thermodynamic activities.

**Table 4:** PC-SAFT pure-component parameters of the components investigated in this work.

Component	$M_{i}/g\;{\mathrm{mol}}^{-1}$	$m_{seg}\;M_{i}^{-1}/\mathrm{mol}\;g^{-1}$	$\sigma_{i}/Å$	$u_{i}\;k_{B}^{-1}/K$	$\epsilon^{A_{i}B_{i}}\;k_{B}^{-1}/K$	$\kappa^{A_{i}B_{i}}$	$N_{i}^{assoc}$	Reference
CA	192.12	0.04448	2.723	227.18	2488	0.044	4/4	[48]
CBZ	236.27	0.04223	2.6583	151.55	1094	0.02	1/1	[18]
GA	132.12	0.03358	2.799	257.67	1762.5	0.02	2/2	[18]
NA	122.12	0.0380	2.1775	176.69	2195.3	0.02	2/2	[49]
SA	118.09	0.0367	3.0546	477.44	1701.69	0.02	2/2	[49]
TP	180.16	0.07607	3.0354	163.97	976.2	0.02	1/1	[50]
urea	60.06	0.07066	2.446	368.23	3068.31	0.001	1/1	[51]
fructose	180.16	0.0410	2.849	237.19	5000	0.1	5/5	[52]
xylitol	152.15	0.0411	2.91	242.24	5000	0.1	5/5	[52]
water	18.02	1.2047	^a^	353.94	2425.7	0.045 ^b^	1/1	[53]

^a^σ = 2.7927 + 10.11 · exp(−0.01775 · T) − 1.417 · exp(−0.01146 · T). ^b^ Typo in reference [53].

**Table 5:** PC-SAFT binary interaction parameters used in this work.

Components	k_ij,T_/K^−1^	k_ij,b_	Reference
CA/water	0	−0.07	[16]
CBZ/water	1.77 × 10^−4^	−0.12745	[19]
GA/water	0	0.00587	this work ^a^
NA/water	9.46 × 10^−5^	−0.0294	[49]
SA/water	−7.3 × 10^−5^	0.00556	[49]
TP/water	−2.40 × 10^−4^	−0.0880	[50]
urea/water	0	−0.0438	[51]
fructose/water	1.40 × 10^−4^	−0.0972	[52]
xylitol/water	2.00 × 10^−4^	−0.109	[52]
CBZ/NA	−1 × 10^−3^	0.2091	[18]
NA/SA	0	0	[26]
TP/CA	0	−0.1125	this work ^b^
urea/GA	0	−0.0464	this work ^a^

^a^ fitted to solubility obtained from Chadwick et al. [27]. ^b^ fitted to solubility obtained from Jayasankar et al. [15].

**Table 6:** Results of the stability measurements after 49 days of storage at a defined RH. Checkmarks indicate that the prepared CC was still present after 49 days of storage and transformation could not be detected. Xs represent the formation of a CC anhydrate or CF as indicated. Circles show samples containing more than 2% water after 49 days of storage. Shaded regions in the table indicate samples/conditions for which deliquescence was predicted.

CC	Add. Crystals	Excipient	RH/%					
			0	58	76	86	93	98
(SA/NA)	SA	**-**	**✓**	**-**	**✓**	**✓**	**✓**	**✓**
(urea/GA 2:1)	-	**-**	**✓**	**-**	**✓**	**✓**	**✓**	**✓**◯
(urea/GA 1:1)	2:1 CC	**-**	**✓**	**-**	**✓**	**✓**	**✓**◯	**✓**◯
(CBZ/NA)	NA	**-**	**✓**	**-**	**✓**	**✓**	**✓**	**✓**◯
(TP/CA) hydrate	CA	**-**	**✓**	**-**	**✘** CC anhydr.	**✓**◯	**✓**◯	**✓**◯
(SA/NA)	SA	fructose	**✓**	**✓**	**✘**◯ NA	**-**	**-**	**-**
(CBZ/NA)	NA	fructose	**✓**	**✓**	**✓**◯	**-**	**-**	**-**
(TP/CA) hydrate	CA	fructose	**✓**	**✓**◯	**✓**◯	**-**	**-**	**-**
(SA/NA)	SA	xylitol	**✓**	**✓**	**✓**	**✓**◯	**-**	**-**
(CBZ/NA)	NA	xylitol	**✓**	**✓**	**-**	**✓**◯	**-**	**-**
(TP/CA) hydrate	CA	xylitol	**✓**	**✓**◯	**✓**◯	**✓**◯	**-**	**-**

**Figure A6:**
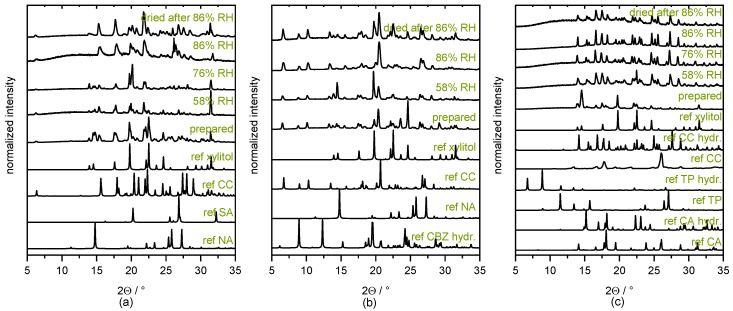
Measured diffractograms of stability measurements of the CC in presence of xylitol: (**a**) SA/NA CC; (**b**) CBZ/NA CC; (**c**) TP/CA CC hydrate. Reference (ref) diffractograms were obtained from the Cambridge Structural Database with following identifiers: SA/NA CC (DUZPAQ), SA (SUCACB03), NA (NICOAM), CBZ/NA CC (UNEZES), CBZ dihydrate (FEFNOT), TP/CA CC hydrate (KIGKAN), TP/CA CC anhydrate (extracted from Jayasankar [59]), TP hydrate (THEOPH), TP anhydrate (BAPLOT07), CA hydrate (CITARC), and CA anhydrate (CITRAC10).

## Data Availability

Data is contained within the article and supplementary material.

## Supplementary Materials

The following are available online at https://www.mdpi.com/1999-4923/13/3/433/s1, Table S1: Prepared composition of API, CF, and solvent for the preparation of CCs, Tables S2–S6: Water sorption of CCs stored at different conditions., Tables S7–S9: Water sorption of CCs in presence of excipient stored at different conditions and the mass fraction of excipient present next to the prepared CC.
