# Supplementary material for: Stability of Pharmaceutical Co-Crystals at Humid Conditions Can Be Predicted
Source: Pharmaceutics. 2021 Mar 23;13(3):433. doi: 10.3390/pharmaceutics13030433 (PMC8004816; doi:10.3390/pharmaceutics13030433)
Supplement: Supplementary file 1 [file pharmaceutics-13-00433-s001.pdf]

pharmaceutics

Supplement Materials: Stability of Pharmaceutical Co-Crystals at Humid Conditions Can Be Predicted

Heiner Veith, Maximilian Zäh, Christian Luebbert, Naír Rodríguez-Hornedo and Gabriele Sadowski

Compositions used to prepare Co-Crystals

Table S1. Prepared composition of API, CF, and solvent for the preparation of CCs.

System	m_{API} / g	m_{CF} / g	$m_{solvent}$ / g
SA/NA/ethanol	1.7795	1.0368	6.9361
CBZ/NA/methanol	0.9876	1.227	5.5891
TP/CA/water	0.7638	4.5011	2.48
Urea/GA/water (2:1 CC)	0.9966	0.8326	3.5508
Urea/GA/water (1:1 CC)	0.7749	3.3812	2.8882

Water sorption of prepared CC samples.

Table S2. Water sorption of SA/NA CC stored at different conditions.

	w_{water}			
storage time / days	76% RH	86% RH	93% RH	98% RH
1	0.009	0.006	0.009	0.012
3	0.006	0.012	0.010	0.012
7	0	0.004	0.004	0.008
21	0.007	0.003	0.016	0.029
49	0.011	0.006	0.002	0.005

Table S3. Water sorption of urea/GA CC (2:1) stored at different conditions.

	w_{water}			
storage time / days	76% RH	86% RH	93% RH	98% RH
1	0.014	0.018	0.022	0.043
3	0	0.021	0.021	0.048
7	0.004	0.009	0.020	0.036
21	0.005	0.017	0.035	0.054
49	0.002	0.016	0.016	0.057

Table S4. Water sorption of urea/GA CC (1:1) stored at different conditions.

	w_{water}			
**storage time / days**	**76% RH**	**86% RH**	**93% RH**	**98% RH**
1	0	0	0.035	0.036
3	0.015	0.005	0.078	0.137
7	0	0	0.068	0.172
21	0.013	0.005	0.077	0.245
49	0	0	0.055	0.195

Table S5. Water sorption of CBZ/NA CC stored at different conditions.

	w_{water}			
**storage time / days**	**76% RH**	**86% RH**	**93% RH**	**98% RH**
1	0	0.007	0.003	0.020
3	0.007	0.013	0	0.031
7	0	0.002	0.007	0.024
21	0.019	0.009	0.007	0.046
49	0	0.002	0.007	0.022

Table S6. Water sorption of TP/CA CC stored at different conditions.

	w_{water}			
**storage time / days**	**76% RH**	**86% RH**	**93% RH**	**98% RH**
1	0.008	0.087	0.129	0.190
3	0.022	0.169	0.253	0.304
7	0.010	0.174	0.240	0.361
21	0.016	0.195	0.246	0.393
49	0.011	0.196	0.242	0.389

Table S7: Water sorption of SA/NA CC in presence of excipient stored at different conditions and the mass fraction of excipient present next to the prepared CC.

additional excipient	storage condition	storage time / days	w_{water}	$w_{excipient}$
fructose	60%	1	0.007	0.35
		3	0.006	0.24
		7	0.012	0.27
		21	0.007	0.25
		49	0.011	0.42
	75%	1	0.073	0.28
		3	0.084	0.29
		7	0.073	0.25
		21	0.081	0.25
		49	0.071	0.24
xylitol	60%	1	0.012	0.25
		3	0.009	0.29
		7	0	0.21
		21	0.000	0.36
		49	0.004	0.25
	75%	1	0.004	0.26
		3	0.013	0.29
		7	0.003	0.32
		21	0	0.26
		49	0.011	0.36
	85%	1	0.056	0.20
		3	0.152	0.25
		7	0.197	0.34
		21	0.156	0.24
		49	0.203	0.33

Table S8. Water sorption of CBZ/NA CC in presence of excipient stored at different conditions and the mass fraction of excipient present next to the prepared CC.

additional excipient	storage condition	storage time / days	w_{water}	$w_{excipient}$
fructose	60%	1	0.007	0.27
		3	0.016	0.27
		7	0.006	0.34
		21	0	0.22
		49	0.016	0.32
	75%	1	0.093	0.33
		3	0.089	0.27
		7	0.111	0.40
		21	0.129	0.40
		49	0.103	0.34
xylitol	60%	1	0.009	0.29
		3	0	0.26
		7	0.010	0.32
		21	0.019	0.32
		49	0.007	0.46
	85%	1	0.069	0.44
		3	0.112	0.37
		7	0.256	0.50
		21	0.211	0.38
		49	0.245	0.44

Table S9. Water sorption of TP/CA CC in presence of excipient stored at different conditions and the mass fraction of excipient present next to the prepared CC.

additional excipient	storage condition	storage time / days	w_{water}	$w_{excipient}$
fructose	60%	1	0.048	0.42

		3	0.053	0.41
		7	0.095	0.40
		21	0.110	0.42
		49	0.099	0.34
	75%	1	0.091	0.42
		3	0.129	0.41
		7	0.164	0.33
		21	0.146	0.32
		49	0.146	0.43
xylitol	60%	1	0.013	0.34
		3	0.016	0.47
		7	0.024	0.37
		21	0.028	0.37
		49	0.076	0.30
	75%	1	0.062	0.43
		3	0.107	0.38
		7	0.144	0.39
		21	0.162	0.41
		49	0.133	0.35
	85%	1	0.137	0.30
		3	0.202	0.22
		7	0.240	0.30
		21	0.257	0.36
		49	0.290	0.49
